# Copper delivery to an endospore coat protein of *Bacillus subtilis*

**DOI:** 10.3389/fcell.2022.916114

**Published:** 2022-09-05

**Authors:** Jaeick Lee, Rosemary A. Dalton, Christopher Dennison

**Affiliations:** Biosciences Institute, Newcastle University, Newcastle Upon Tyne, United Kingdom

**Keywords:** copper, copper storage, bacterial copper homeostasis, bacterial laccases, sporulation, *Bacillus subtilis*

## Abstract

A family of cytosolic copper (Cu) storage proteins (the Csps) bind large quantities of Cu(I) via their Cys-lined four-helix bundles, and the majority are cytosolic (Csp3s). The presence of Csp3s in many bacteria appears inconsistent with the current dogma that bacteria, unlike eukaryotes, have evolved not to maintain intracellular pools of Cu due to its potential toxicity. Sporulation in *Bacillus subtilis* has been used to investigate if a Csp3 binds Cu(I) in the cytosol for a target enzyme. The activity of the Cu-requiring endospore multi-Cu oxidase *Bs*CotA (a laccase) increases under Cu-replete conditions in wild type *B. subtilis*. In the strain lacking *Bs*Csp3 lower *Bs*CotA activity is observed and is unaffected by Cu levels. *Bs*Csp3 loaded with Cu(I) readily activates apo-*Bs*CotA *in vitro*. Experiments with a high affinity Cu(I) chelator demonstrate that Cu(I) transfer from Cu(I)-*Bs*Csp3 must occur via an associative mechanism. *Bs*Csp3 and *Bs*CotA are both upregulated during late sporulation. We hypothesise that *Bs*Csp3 acquires cuprous ions in the cytosol of *B. subtilis* for *Bs*CotA.

## Introduction

**FIGURE 1:**
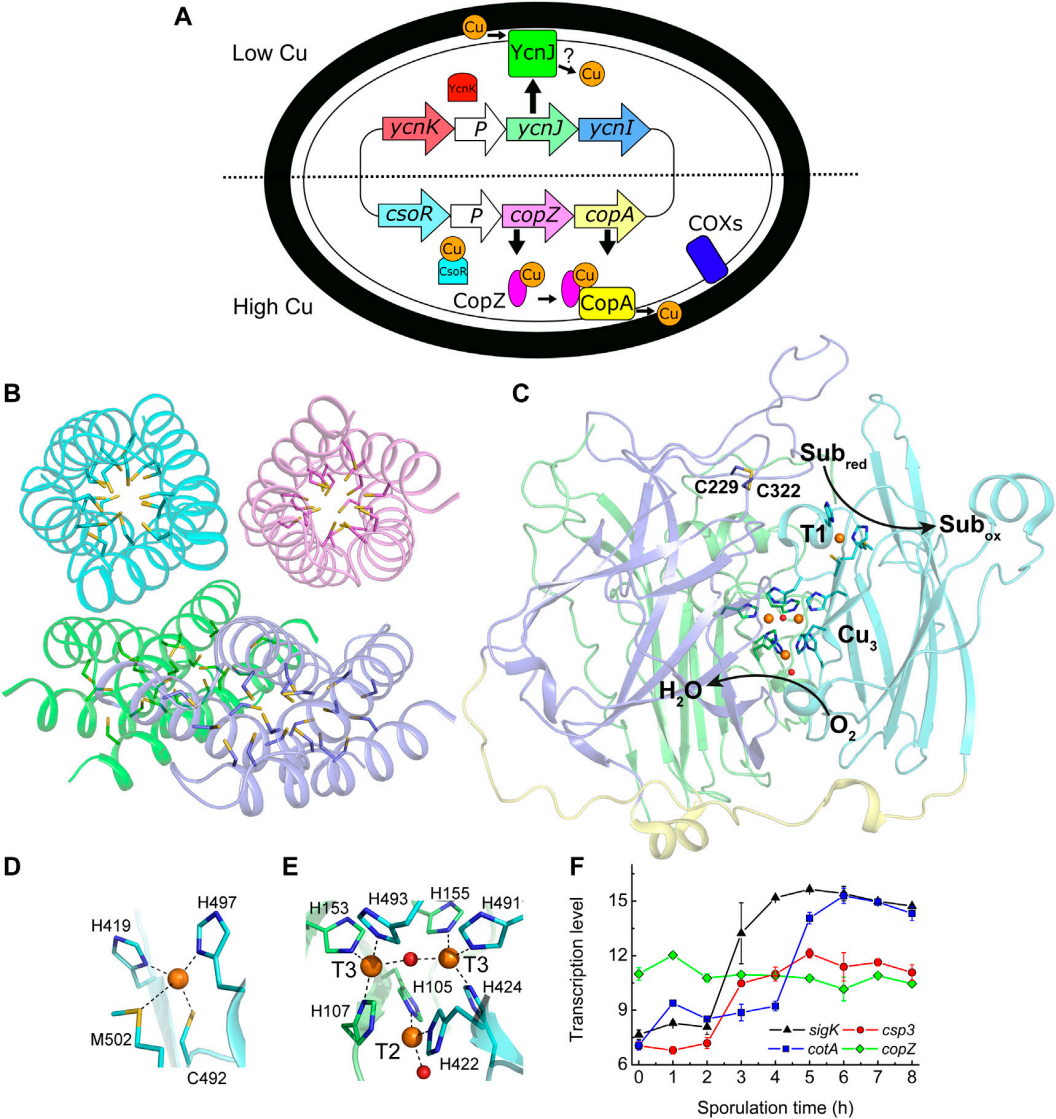
Copper handling, a cytosolic Cu(I) storage protein, a Cu-requiring enzyme, and their transcription during sporulation in *B. subtilis*. **(A)** An overview of Cu homeostasis in *B. subtilis* including Cu (orange circles, oxidation state undefined) export by CopA and CopZ (regulated by CsoR) (Smaldone and Helmann 2007), and import by YcnJ (regulated by YcnK) (Chillappagari et al., 2009; Hirooka et al., 2012). YcnI is membrane bound and binds Cu(II) *in vitro*, but its role in Cu homeostasis is unclear (Damle et al., 2021). The only currently known Cu-requiring enzymes in vegetative *B. subtilis* cells are two cytochrome oxidases (COXs) located on the plasma membrane (Lauraeus et al., 1991). **(B)** The crystal structure of Cu(I)-free *Bs*Csp3 (PDB: 5FIG), a tetramer of four-helix bundles each with 19 Cys residues pointing into their cores enabling the binding of up to ∼20 Cu(I) ions per monomer (Vita et al., 2016). **(C)** The crystal structure of the endospore multi-Cu oxidase (a laccase) *Bs*CotA (PDB: 1GSK, Enguita et al., 2003) with domains 1, 2, and 3 coloured green, slate and cyan, respectively (the linking regions are yellow). Substrates are oxidized (Sub_red_ to Sub_ox_) at the T1 Cu centre with electrons passed to the T2/T3 trinuclear (Cu_3_) cluster where oxygen is reduced to water. Also highlighted is the disulfide bond between Cys229 and Cys322. Detailed views of the T1 Cu site **(D)** and the Cu_3_ cluster **(E)** are shown. The side chains of coordinating residues are represented as sticks, Cu ions as orange spheres and the oxygen atoms of water (bound to the T2 Cu) and hydroxide (bridging the T3 Cu ions) ligands as red spheres in **(C–E)**. **(F)** Transcription profiles (Nicolas et al., 2012) of the *sigK* (σ^K^, which facilitates expression of outer and inner spore coat proteins, black triangles), *csp3* (red circles), *cotA* (blue squares) and *copZ* (green diamonds) genes during sporulation.

Copper (Cu) is essential for most organisms, but use of this metal ion is associated with significant risks due to its potential toxicity. The availability of Cu is regulated by the presence of high-affinity sites in both eukaryotes (Rae et al., 1999) and prokaryotes (Changela et al., 2003). Therefore, all intracellular Cu(I) is tightly bound to either proteins or small molecules, i.e. there is no ‘free’ Cu(I) (Rae et al., 1999; Changela et al., 2003; Festa and Thiele 2011). Import, cytosolic handling, trafficking to different locations, and storage of Cu have all been characterised in eukaryotic cells (Festa and Thiele 2011). In bacteria, some of these processes are either not thought to occur, or are not yet fully understood. For example, the plasma membrane protein CcoG, which reduces Cu(II) to the preferred intracellular oxidation state [Cu(I)] has only recently been identified in bacteria as a cytochrome oxidase (COX) assembly factor (Marckmann et al., 2019). The reduction of Cu(II) prior to import into eukaryotic cells has been known to happen for many years (Hassett and Kosman 1995; Festa and Thiele 2011). Excess Cu(I) is removed from the cytosol by probably the best-studied component of bacterial Cu homeostasis (homologues are present in eukaryotes); a Cu-transporting P-type ATPase (CopA), which can be assisted by the cytosolic Cu metallochaperone CopZ (Figure 1A) (Rensing et al., 2000; Solioz et al., 2010; Festa and Thiele 2011; Rensing and McDevitt 2013; Meydan et al., 2017). The toxicity of Cu can involve Cu(I) binding in place of the native metal in cytosolic iron-sulfur (Fe-S) cluster-containing proteins (Macomber and Imlay 2009), and Cu catalyses ROS formation (Solioz et al., 2010; Festa and Thiele 2011; Rensing and McDevitt 2013). The intracellular damage that Cu causes, and the current dearth of intracellular Cu-requiring enzymes (Ridge et al., 2008), has resulted in a prevailing view that bacteria have evolved not to use this metal ion in the cytosol (Ridge et al., 2008; Rensing and McDevitt 2013). However, there is no *a priori* reason why bacteria, like eukaryotes, cannot utilise Cu in this compartment if mechanisms are available to enable its safe handling, i.e., by ensuring tight chelation and specific delivery. The presence of cytosolic Cu storage proteins (Csps) that bind large quantities of Cu(I) with high affinity (Vita et al., 2015; Vita et al., 2016; Dennison et al., 2018; Lee and Dennison 2019) provide a possible route for intracellular Cu use in bacteria.

The Csps were first identified in Gram-negative bacteria that oxidize methane (Vita et al., 2015). These methanotrophs can possess different Csp homologues, all having many Cys residues lining the cores of their four-helix bundles that enable the binding of a large number of Cu(I) ions (Vita et al., 2015; Vita et al., 2016; Dennison et al., 2018). A Csp exported from the cytosol (Csp1) stores up to 52 Cu(I) ions per tetramer for the particulate (membrane-bound) methane monooxygenase (pMMO) in the model methanotroph *Methylosinus trichosporium* OB3b (*Mt*Csp1) (Vita et al., 2015). *Mt*Csp1 is upregulated at the Cu concentrations required for methane oxidation by pMMO in switchover methanotrophs (Gu and Semrau 2017), which uses a soluble Fe MMO when Cu is limiting (DiSpirito et al., 2016). However, a cytosolic Csp homologue (*Mt*Csp3) is not upregulated with pMMO in *M. trichosporium* OB3b (Gu and Semrau 2017).

The Gram-positive bacterium *Bacillus subtilis* is an ideal model system for investigating the role of a Csp3 as its Cu homeostasis system is well characterised (Figure 1A) (Radford et al., 2003; Smaldone and Helmann 2007; Chillappagari et al., 2009; Ma et al., 2009; Solioz et al., 2010; Hirooka et al., 2012; Damle et al., 2021). This includes the *copZA* operon (Cu efflux machinery, *vide supra*) and its Cu-sensing repressor CsoR (Radford et al., 2003; Smaldone and Helmann 2007; Ma et al., 2009; Solioz et al., 2010). The membrane protein YcnJ is upregulated under Cu-limiting conditions, controlled by the suggested repressor YcnK (Chillappagari et al., 2009; Hirooka et al., 2012), and has been proposed to play a role in Cu acquisition (Figure 1A). The gene for the membrane-anchored YcnI is part of the same (*ycnKJI*) operon and is also thought to be regulated by YcnK (Hirooka et al., 2012). The soluble domain of YcnI binds Cu(II) *in vitro*, and this protein has been suggested to function as a Cu metallochaperone (Damle et al., 2021. Cytosolic Cu(I) could be safely stored by the *B. subtilis* Csp3 homologue (YhjQ, herein *Bs*Csp3) whose core is lined with 19 Cys residues (Figure 1B), enabling the binding of ∼80 Cu(I) ions per tetramer *in vitro* (Vita et al., 2016).

Only two families of Cu enzymes are currently known to be present in *B. subtilis*; two COXs (one without the Cu_A_ site in subunit II), located on the plasma membrane (Figure 1A) and the multi-Cu oxidase (MCO; a laccase) *Bs*CotA (Lauraeus et al., 1991; Hullo et al., 2001; Martins et al., 2002; Enguita et al., 2003). Assembly of the COXs, including their acquisition of Cu has been extensively studied (for example von Wachenfeldt et al., 2021). *Bs*CotA is an outer spore-coat (endospore) enzyme (McKenney et al., 2013) that possesses the typical type 1 (T1), 2 (T2) and 3 (T3) Cu sites of an MCO (Enguita et al., 2003), which are all involved in the catalytic cycle (see Figures 1C–E). *Bs*CotA produces a melanin-like pigment thought to provide spores with protection against hydrogen peroxide and UV light (Hullo et al., 2001; McKenney et al., 2013). This enzyme is upregulated during the latter stages of sporulation, as is *Bs*Csp3 (Figure 1F) (Nicolas et al., 2012).

Herein we demonstrate that, despite previous preliminary work from our laboratory (Vita et al., 2016), *Bs*Csp3 does not provide resistance to toxicity caused by elevated Cu levels in *B. subtilis*. We have therefore tested the hypothesis that *Bs*Csp3 binds Cu(I) ions in the cytosol for a Cu-requiring enzyme by investigating the effect of gene deletion on the activity of *Bs*CotA in spores grown under Cu limiting and replete conditions. The data obtained indicate a role for *Bs*Csp3 in ensuring maximum *Bs*CotA activity. The ability of Cu(I)-*Bs*Csp3 to activate apo-*Bs*CotA has been confirmed *in vitro*. A model for how *Bs*CotA is loaded with Cu during sporulation is proposed. This is the first example showing an enzyme acquiring Cu(I) in the cytosol of a bacterium, as well as identifying the protein from which the metal ion is obtained.

## Results

### *Bs*Csp3 is not required in combating Cu toxicity in *B. subtilis*

The presence of *Bs*Csp3 with a high capacity for Cu(I) in the cytosol of *B. subtilis* (Vita et al., 2015; Vita et al., 2016; Dennison et al., 2018) would suggest a role in helping to prevent the issues associated with excess Cu (Macomber and Imlay 2009; Lee and Dennison 2019). The toxicity of Cu to bacteria is highlighted by how increasing Cu concentrations limited the growth of wild type (WT) *B. subtilis* in LB medium (Supplementary Figure S1). At ≥1.5 mM Cu cells started to grow more slowly, with a very small increase in the absorbance/OD observed only after more than 6 h at 2 mM added Cu, coinciding with elevated intracellular Cu concentrations (Supplementary Figure S2). Very similar growth and Cu accumulation results were obtained (Supplementary Figures S1, S2) for the *B. subtilis* strain (Δ*csp3*) lacking the *csp3* gene (*yhjQ*). The growth studies reported herein demonstrate that *Bs*Csp3 is not solely required in helping prevent the harmful effects of elevated Cu levels on *B. subtilis* (more details are provided in the legend to Supplementary Figure S1). Therefore, the protein apparently does not have a function like the eukaryotic cytosolic Cys-rich metallothioneins (Festa and Thiele 2011).

### Using sporulation to determine the function of *Bs*Csp3

**FIGURE 2:**
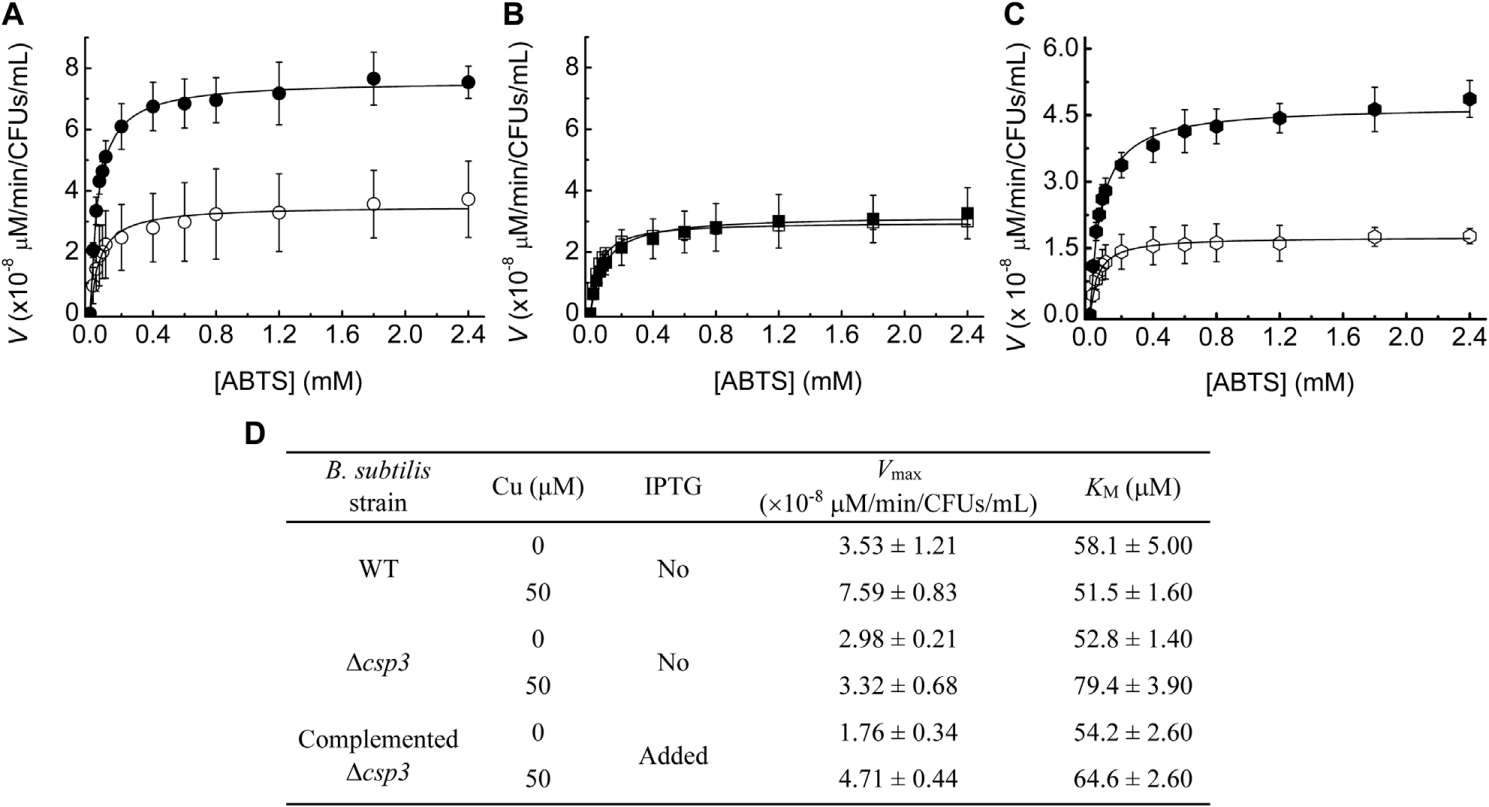
The influence of Cu levels and *Bs*Csp3 on *Bs*CotA activity in *B. subtilis* spores. Michaelis-Menten plots of *Bs*CotA activity for heated purified spores from WT **(A)**, Δ*csp3* **(B)**, and the complemented Δ*csp3* **(C)** strains. Spores **(A,B)** were produced in DSM plus no (open symbols) and 50 μM (black filled symbols) added Cu(NO_3_)_2_. For the complemented Δ*csp3* strain **(C)** sporulation was carried out in the presence of 1 mM IPTG with either no (open symbols) or 50 μM (black filled symbols) added Cu(NO_3_)_2_. Plots from which the initial rates for WT and Δ*csp3 B. subtilis* spores were obtained are shown in Supplementary Figure S3. Kinetics measurements were made in 100 mM citrate-phosphate buffer pH 4.0 at 37°C using three different sets of spores (averages and standard deviations are shown). **(D)** The *V*_max_ and *K*_M_ values obtained from the fits of the data in **(A–C)** to the Michaelis-Menten equation. The *K*_M_ values for the oxidation of ABTS by *Bs*CotA in spores are all in the range of those reported for purified enzyme (Martins et al., 2002; Durão et al., 2008; Fernandes et al., 2011).

*Bs*CotA, along with the two COXs (whose Cu acquisition is well-characterised, von Wachenfeldt et al., 2021), are the only known Cu-requiring enzymes present in spores. The *csp3* and *cotA* genes are both upregulated (Nicolas et al., 2012) at similar stages during sporulation (Figure 1F). We have therefore studied whether *Bs*Csp3 is involved in Cu(I) supply to *Bs*CotA. This enzyme binds four Cu ions (Figures 1C–E), which are required to enable oxidation of the laccase substrate 2,2′-azino-bis(3-ethylbenzothiazoline-6-sulfonic acid) (ABTS) *in vitro* and in spores (Martins et al., 2002). We have used the oxidation of ABTS to assess the relative amounts of Cu-*Bs*CotA in *B. subtilis* spores (Figure 2). For WT *B. subtilis* spores, the ability to oxidise ABTS increased approximately two-fold when 50 μM Cu is added to Difco sporulation medium (DSM) (Figures 2A,D, Supplementary Figures S3A,B). This indicated that unless supplemented, DSM does not contain sufficient Cu (the Cu concentration in DSM without any added Cu is ∼0.4 μM) to allow all of the *Bs*CotA produced during sporulation to be active.

The *Bs*CotA activity of Δ*csp3 B. subtilis* spores grown in DSM without added Cu was similar to that for WT spores produced under the same conditions (Figures 2B,D, Supplementary Figures S3C,D). However, unlike for WT *B. subtilis*, supplementing DSM with Cu during sporulation had no effect on *Bs*CotA activity for the Δ*csp3* strain. These results indicate that *Bs*Csp3 plays a role in Cu acquisition by *Bs*CotA during sporulation, particularly under Cu-replete conditions. Some *Bs*CotA activity remained for Δ*csp3 B. subtilis* spores, and an alternative mechanism of Cu acquisition by *Bs*CotA must exist, which could also be responsible for the activity observed in the WT strain under Cu-limiting conditions.

To confirm that *Bs*Csp3 is involved in Cu(I) supply to *Bs*CotA, the Δ*csp3* strain was complemented by introducing the *csp3* gene at a different location (the *amyE* locus), which can be induced by isopropyl β-D-thiogalactopyranoside (IPTG). The highest *Bs*CotA activity was obtained for spores of this strain grown in the presence of IPTG and Cu (Figures 2C,D). Under these conditions, activity was almost three-fold greater than without their addition, similar to the increase for WT *B. subtilis* spores under Cu-replete conditions (Figures 2A,D).

### The activation of Cu-free-*Bs*CotA by Cu(I)-*Bs*Csp3 *in vitro*

**FIGURE 3:**
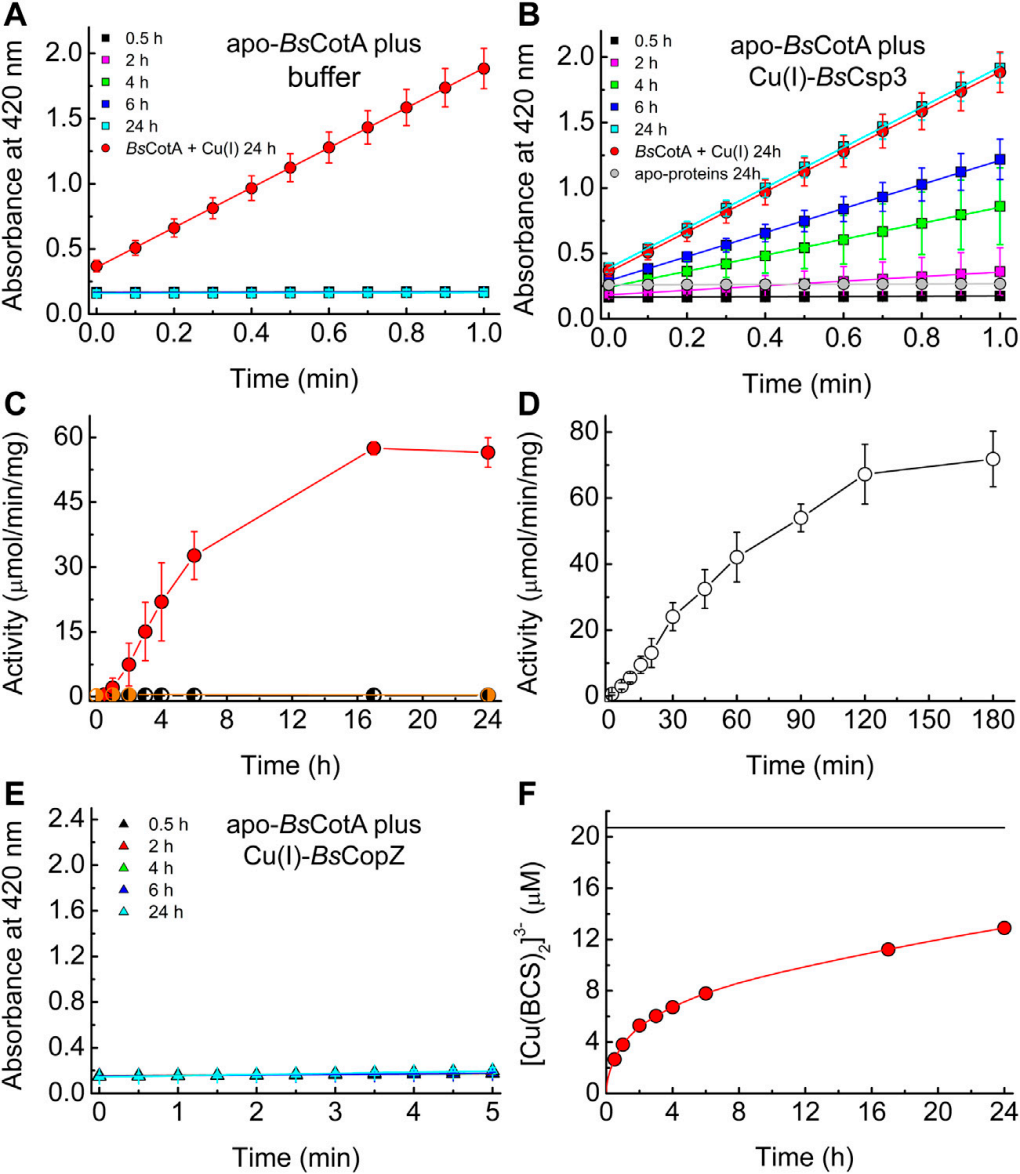
The activation of apo-*Bs*CotA by Cu(I)-*Bs*Csp3 and associative Cu(I) transfer. Plots of absorbance at 420 nm against time for the reaction with 2.4 mM ABTS (at 37°C) of mixtures of apo-*Bs*CotA incubated with buffer **(A)** and Cu(I)-*Bs*Csp3 **(B)** for up to 24 h. Also shown is the data obtained when apo-*Bs*CotA was incubated with Cu(I) (red circles in A and B) and apo-*Bs*Csp3 (grey circles in B) for 24 h. Mixtures were incubated under anaerobic conditions (apart from the reaction with apo-*Bs*Csp3) in 20 mM 4-(2-hydroxyethyl)piperazine-1-ethanesulfonic acid (HEPES) pH 7.5 plus 200 mM NaCl (the buffer used in **A**), and *Bs*CotA activity was measured in 100 mM citrate-phosphate buffer pH 4.0. **(C)** Plots of activity against incubation time of apo-*Bs*CotA plus buffer alone (half-black circles), Cu(I)-*Bs*Csp3 (red circles), and apo-*Bs*Csp3 (half-grey circles) for up to 24 h (very similar values to those at 24 h were measured at 48 h for buffer and Cu(I)-*Bs*Csp3). In **(D)** are activity data obtained when apo-*Bs*CotA with the Cys229-Cys322 disulfide bond reduced, was mixed with Cu(I)-*Bs*Csp3 for up to 180 min. **(E)** Plots of absorbance at 420 nm against time for the reaction with 2.4 mM ABTS (at 37°C) of apo-*Bs*CotA incubated with Cu(I)-*Bs*CopZ for up to 24 h (there was also no sign of activity after 48 h). All activity data are averages from three to six independent experiments (apart from apo-*Bs*CotA plus apo-*Bs*Csp3) with error bars showing standard deviations. **(F)** A plot of [Cu(BCS)_2_]^3-^ concentration against time for *Bs*Csp3 (1.08 µM) plus 18.0 equivalents of Cu(I) mixed with 2.5 mM BCS (red line) carried out in the same buffer as that used in **(A–E)** under anaerobic conditions. The data at 0.5, 1, 2, 3, 4, 6, 17, and 24 h, which correspond to times at which *Bs*CotA activity was measured **(C)**, are shown by red circles. The black line indicates the outcome of the same experiment but with 6.64 M guanidine-HCl present in the buffer. The Cu(I)-protein unfolds resulting in much faster removal of cuprous ions, giving the end point for the reaction (the value shown was obtained after 2 h). The average percentage removal compared to that for unfolded samples, and the standard deviations, from three independent experiments are listed in Supplementary Table S1.

The above data support the hypothesis that *Bs*Csp3 binds Cu(I) in the cytosol under Cu-replete conditions, which is used to metallate *Bs*CotA. The ability of Cu(I)-*Bs*Csp3 to activate apo (Cu-free)-*Bs*CotA was therefore studied *in vitro* (Figures 3A–D). Apo-*Bs*CotA is inactive (Figures 3A,C), whilst the addition of Cu produces enzyme that rapidly oxidises ABTS (for example see Figure 3A and Martins et al., 2002). Cu(I)-*Bs*Csp3 readily activates apo-*Bs*CotA (Figures 3B,C), giving similar reactivity to enzyme plus Cu(I) at 24 h (Figure 3B). This is consistent with Cu(I) transfer from Cu(I)-*Bs*Csp3 to apo-*Bs*CotA (apo-*Bs*Csp3 does not activate apo-*Bs*CotA, see Figures 3B,C), and >50% activation is achieved in 6 h (Figure 3C). The number of free thiols in this form of *Bs*CotA, which possess four Cys residues, was routinely determined to be ∼2, consistent with the Cys229-Cys322 disulfide bond being present in the overexpressed enzyme purified from *E. coli* (Enguita et al., 2003). Reduction of the protein with dithiothreitol (DTT) resulted in ∼3.5 free thiols and thus cleavage of the Cys229-Cys322 disulfide. Reduced apo-*Bs*CotA reacts much more rapidly with Cu(I)-*Bs*Csp3 and >50% activation is achieved in just over 45 min (Figure 3D). Inactive apo-*Bs*CotA was found to contain no detectable Cu (<0.2 equivalents) by atomic absorption spectrometry (AAS). After transfer, 4.11 ± 0.75 (*n* = 4) equivalents were bound, and when measured the absorbance values at 600 and 330 nm were consistent with full occupancy of the T1 and T3 sites with Cu(II), respectively (Durão et al., 2008). Another cytosolic Cu(I)-binding protein with a well-established role in Cu homeostasis (delivering Cu(I) to *Bs*CopA, Radford et al., 2003) and a similar Cu(I) affinity (∼10^17^ M^−1^ at pH 7.5) to *Bs*Csp3 (Badarau and Dennison 2011a; Vita et al., 2016) is *Bs*CopZ (see Figure 1A). After incubation of apo-*Bs*CotA with Cu(I)-*Bs*CopZ for 24 h almost no activity is observed (Figure 3E), indicating Cu(I) transfer does not occur. A large excess of bathocuproine disulfonate (BCS) removes only ∼60% of Cu(I) from *Bs*Csp3 in 24 h (Figure 3F; Supplementary Table S1). The slow kinetics for this reaction demonstrates that Cu(I) does not freely dissociate from *Bs*Csp3, otherwise the [Cu(BCS)_2_]^3-^ complex would rapidly form. We therefore assume associative mechanisms for the reactions of Cu(I)-*Bs*Csp3 with BCS and also with apo-*Bs*CotA, with the partners interacting prior to Cu(I) transfer.

## Discussion

In this study we have demonstrated that *Bs*Csp3 binds cytosolic Cu(I) and plays a role in supplying Cu(I) to the Cu-requiring enzyme *Bs*CotA during sporulation (Figure 2). This is not the only mechanism available to load *Bs*CotA with Cu as some activity is observed in Δ*csp3 B. subtilis*. A possibility we considered was that the cytosolic Cu metallochaperone *Bs*CopZ, as well as transferring Cu(I) to *Bs*CopA (Figure 1A), may supply cuprous ions to *Bs*CotA. The *in vitro* studies reported here show that despite the similar Cu(I) affinity to *Bs*Csp3 (Badarau and Dennison 2011a; Vita et al., 2016), Cu(I)-*Bs*CopZ cannot activate apo-*Bs*CotA (Figure 3E), consistent with *Bs*CopZ not being upregulated during sporulation (Figure 1F). Furthermore, *Bs*CotA activity in Δ*csp3 B. subtilis* spores is unaffected when the Cu concentration is higher, conditions which would increase *Bs*CopZ expression. A similar level of activity is determined for WT spores without supplementing DSM with Cu, conditions under which *Bs*CopZ will not be upregulated. Collectively, these data exclude a potential role for *Bs*CopZ in activating *Bs*CotA. The source(s) of Cu(I) for activating *Bs*CotA in the absence of *Bs*Csp3, and also at lower intracellular concentrations of the metal ion, remain(s) to be established. Regardless, the lack of activation of apo-*Bs*CotA by Cu(I)-*Bs*CopZ *in vitro* highlights the specificity of activation by Cu(I)-*Bs*Csp3. This is essential in a cell as it ensures Cu(I) is delivered to where it is needed, as observed for other Cu-homeostasis proteins (Pufahl et al., 1997; Rae et al., 1999; Schmidt et al., 1999; Lamb et al., 2001; Banci et al., 2006; Banci et al. 2010; Banci et al. 2011; Sala et al., 2019).

The high Cu(I) affinity (Vita et al., 2016) of *Bs*Csp3 (1.5 × 10^17^ M^−1^), and the slow formation of [Cu(BCS)_2_]^3-^ when BCS is added to protein fully loaded with Cu(I) (Figure 3F), indicates the transfer of cuprous ions from Cu(I)-*Bs*Csp3 to apo-*Bs*CotA has to occur via an associative mechanism (unassisted Cu(I) off-rates for *Bs*Csp3 can be estimated to be ∼10^–9^ s^−1^, Dennison et al., 2018). This is consistent with the requirement for no intracellular ‘free’ Cu(I). For the acquisition of such tightly bound Cu(I) to be possible, metalation must take place once *Bs*CotA has at least partially folded so the sites where Cu is required have formed. The T1 Cu site is closest to the surface, with its His497 ligand solvent exposed, and is ∼12.5–15.5 Å from the Cu_3_ cluster (Figure 1C). Therefore, *Bs*Csp3 association at more than one location may be required to metalate all of the sites in folded *Bs*CotA. Published Cu(I) affinities of T1 Cu sites (Badarau and Dennison 2011b; North and Wilcox 2019) are (2.1–4.0) × 10^17^ M^−1^, similar to the average Cu(I) affinity of *Bs*Csp3 (Vita et al., 2016). Although Cu(I) affinities are not available for the Cu_3_ cluster, Cu(I) transfer from the storage protein to the enzyme should not be hindered thermodynamically (Banci et al., 2010; Badarau and Dennison 2011b). To facilitate access to the more buried Cu_3_ cluster (required for activity) the protein may need to be partially unfolded. The MCO CueO from *E. coli* undergoes a transition from an ‘open’ non-metallated folded form with accessible Cu sites, to a more ‘closed’ conformation after Cu has bound (Strolle et al., 2016). *Bs*CotA has a disulfide bond between Cys229 and Cys322 (Figure 1C) that is ∼12–13 Å from the T1 Cu site and 17–23 Å from the Cu_3_ cluster (Enguita et al., 2003). Only two of the four Cys residues are reactive in *Bs*CotA overexpressed and purified from *E. coli* (see Materials and Methods) and this form of the enzyme possesses the Cys229-Cys322 disulfide. *In vitro* activation is significantly faster in the absence of the disulfide (Figures 3C,D), and the formation of this bond in *Bs*CotA may be linked to Cu(I) binding *in vivo* (*vide infra*). This is consistent with previous studies that found an increase in the rate of Cu(II) binding when this disulfide was removed (Fernandes et al., 2011), but with limited influence on overall structure and stability.

**FIGURE 4:**
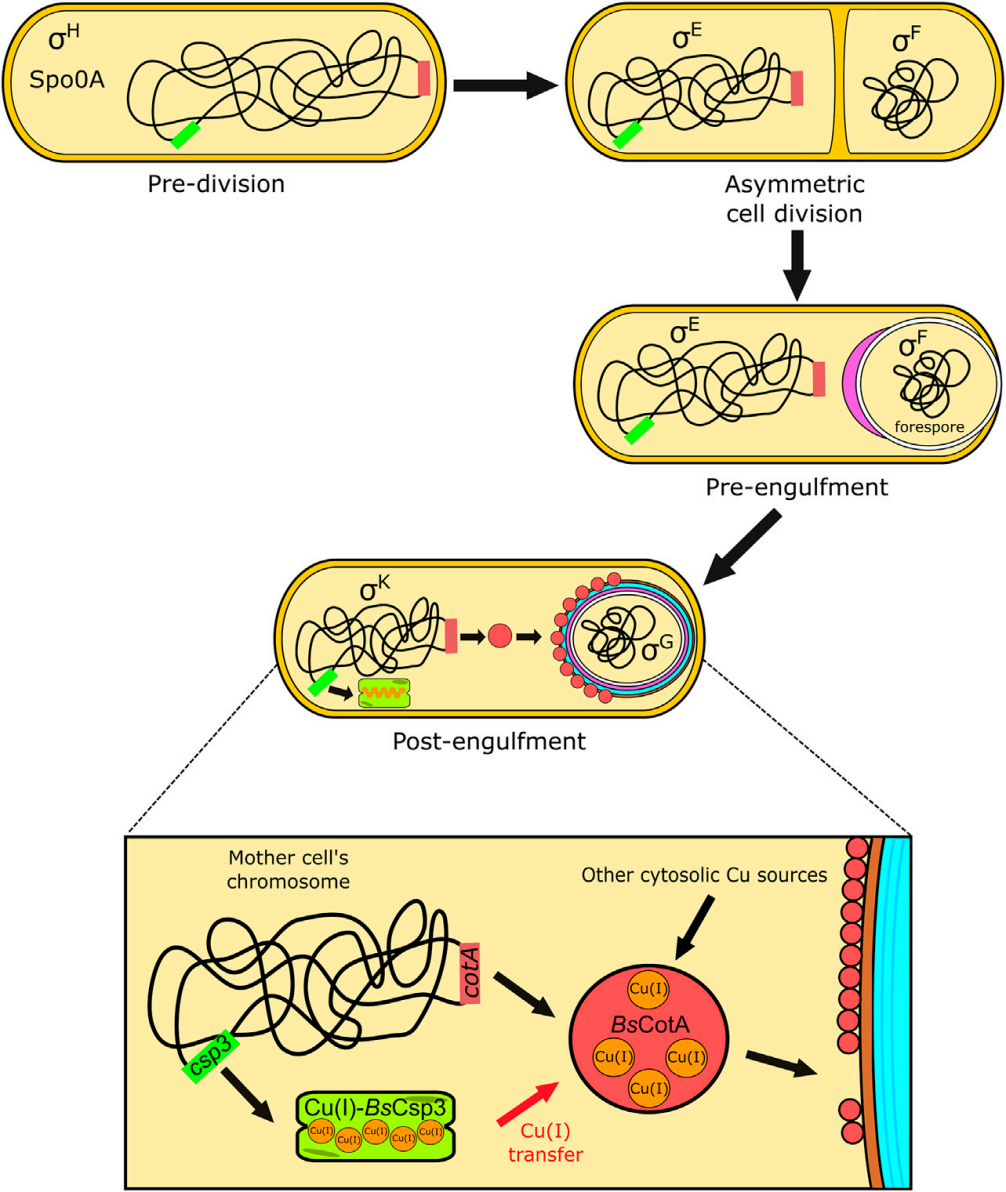
The proposed role of *Bs*Csp3 in Cu(I) acquisition by *Bs*CotA during sporulation in *B. subtilis*. The transcription factor Spo0A, along with σ^H^, initiates sporulation. A septum asymmetrically divides the cell into the forespore and mother cell, with σ^E^ and σ^F^, respectively, activated within these. The mother cell begins engulfment of the forespore and σ^E^ directs gene expression and initiation of spore coat (purple) formation. The expression of *Bs*Csp3 and *Bs*CotA now begins, promoted by σ^K^ (see Figure 1F) and coat assembly continues. We propose that *Bs*Csp3 acquires Cu(I) during this stage of sporulation, which is transferred to *Bs*CotA prior to insertion of the Cu-enzyme into the spore coat.

*Bs*Csp3 and *Bs*CotA expression are regulated by sigma factor K (SigK or σ^K^), which is produced after the forespore has been engulfed by the mother cell (Figure 4). It appears the *csp3* gene constitutes an operon with *yhjR*, an inner spore coat protein (Hosan et al., 2006) also regulated by SigK (Eichenberger et al., 2004) and co-expressed with Csp3 (Nicolas et al., 2012). Upregulation of the *csp3* gene happens prior to *cotA* (Figure 1F), which would allow *Bs*Csp3 to acquire Cu(I) before production of the enzyme requiring the metal. We propose that Cu(I) is transferred to *Bs*CotA before it localizes to the spore coat. If Cu(I) acquisition occurs once *Bs*CotA is part of the spore coat it is possible that YhjR plays a role in assisting this process. As discussed, *Bs*CotA activation by Cu(I)-*Bs*Csp3 *in vitro* is significantly faster in the absence of the Cys229-Cys322 disulfide, indicating that Cu(I) is acquired prior to the formation of this bond *in vivo*. Currently, there is only one known example of Cu acquisition by an enzyme from a partner protein in the cytosol. This is the eukaryotic Cu,Zn-superoxide dismutase (SOD1), which obtains Cu(I) from the Cu metallochaperone CCS (Rae et al., 1999; Schmidt et al., 1999; Lamb et al., 2001; Banci et al., 2010; Banci et al., 2011; Sala et al., 2019; Culotta et al., 1997; Wong et al., 2000). After many years of study, the activation of SOD1 by CCS is now fully understood and has been found to be linked to the formation of an essential disulfide bond in SOD1.

Added importance to understanding the correct metalation of *Bs*CotA is provided by the observation that melanin production interferes with the phagocytosis of pathogenic yeast, and is required to allow survival in macrophages (Eisenman and Casadevall 2012). The related pigment made by Cu-loaded *Bs*CotA is important for spore survival (Hullo et al., 2001; McKenney et al., 2013), and this may include within a host. *Bacillus* spores, and particularly those from *B. cereus*, cause food poisoning and are a common contaminant in a range of foods (Soni et al., 2016; Jessberger et al., 2020). The development of more effective inactivation approaches requires a better understanding of enzymes such as CotA that help protect spores. This includes establishing how they acquire essential cofactors including Cu ions.

## Materials and methods

### WT *B. subtilis* and the strain with the *csp3* gene deleted

WT *B. subtilis* 168 (genotype: *trpC2*) and the strain with the *yhjQ* gene deleted (genotype: *trpC2 ΔyhjQ::erm*, referred to herein as Δ*csp3*) strains were obtained from the *Bacillus* Genetic Stock Centre (BGSC) library (BGSCID 1A1 and BKE10600, respectively). These strains were checked by PCR (Supplementary Figures S4A,B, S5A; Supplementary Table S2). The disrupted *csp3* gene was amplified by PCR using genomic DNA from the Δ*csp3* strain with primers that hybridise ∼300 bp upstream and downstream of this region (Supplementary Figures S4B, S5A; Supplementary Table S2). The resulting fragment was sequenced with primers designed to hybridise ∼20 bp from the ends of the PCR product (Supplementary Table S2) and matches that of the erythromycin resistance gene.

### Growth curves for WT and Δ*csp3 B. subtilis* at increasing Cu concentrations

To test the influence of Cu on WT and Δ*csp3* strains, cultures were grown (agitation at 250 rpm) in LB medium at 37°C overnight, diluted 100-fold in LB and LB plus added Cu(NO_3_)_2_ (0.5–2.0 mM). The absorbance at 600 nm was measured at regular intervals for up to 12 h, and also after 24 h. Cells ( ~  35 ml) were collected and washed, including with buffer plus 10 mM ethylenediaminetetraacetic acid (EDTA) (Lee and Dennison 2019), and digested in 200 μl of 65% HNO_3_ (Ultrapur) for up to 3 days at room temperature. These mixtures were centrifuged at 12,000 g for 10 min, diluted in MilliQ water to give a final HNO_3_ concentration of 2% and analysed for Cu by AAS. The Cu concentration in the stock solution used for these studies was regularly determined by AAS, as described previously (Lee and Dennison 2019).

### The construction of the Δ*csp3 B. subtilis* strain complemented with *csp3*

To insert the *csp3* gene plus its ribosome binding site (RBS) into the *amyE* locus of the Δ*csp3* strain, a region including an additional 28 bp at the 5′ end was amplified from WT *B. subtilis* 168 genomic DNA by PCR using primers; rbs_BsCsp3-F and rbs_BsCsp3-R (Supplementary Figures S4A, S5A; Supplementary Table S2). The product was cloned into pGEM-T (Promega) and the resulting *rbs_csp3* fragment sub-cloned into pDR111, which possesses the IPTG-inducible *P*_*hyerspank*_ promoter (Quisel et al., 2001; Britton at al. 2002), using HindIII and NheI to generate pDR111_rbs_csp3. To obtain a strain possessing an IPTG-inducible copy of the *csp3* gene (*trpC2 ΔyhjQ::erm amyE::P*_*hyspank*_ *-rbs_yhjQ*, called complemented Δ*csp3* herein), Δ*csp3 B. subtilis* was transformed with pDR111_rbs_csp3. Selection was achieved using spectinomycin (50 μg/ml) and successful integration into the chromosomal *amyE* (α-amylase) gene identified by growing on LB agar containing 1% starch and staining with iodine (Engman et al., 2012). Insertion of the *csp3* gene was determined as described above (Supplementary Figures S4C, S5B; Supplementary Table S2), with the location and size of the fragment incorporated confirmed by PCR using the primers pDR111_int-F and pDR111_int-R (Supplementary Figures S4C, S5B; Supplementary Table S2).

### The production of *B. subtilis* spores

WT, Δ*csp3* and complemented Δ*csp3* strains were grown overnight (agitation at 250 rpm) in 20 ml of DSM. Cultures were diluted 50-fold into 200 ml of DSM in a single 1 L Erlenmeyer flask and grown until the absorbance at 580 nm reached ∼0.5. This culture was split into four 50 ml cultures, each in a 250 ml Erlenmeyer flask, and 50 μM Cu(NO_3_)_2_ and 1 mM IPTG added when required. The cultures were grown (agitation at 250 rpm) for 48 h at 37°C and absorbance values at 580 nm measured at regular intervals. To purify spores (Tavares et al., 2013) cultures were centrifuged (4°C) for 10 min at 5,000 g, pellets re-suspended in 50 mM tris(hydroxymethyl)aminomethane (Tris) pH 7.2 plus 50 μg/ml lysozyme and incubated at 37°C for 1 h. After incubation and further centrifugation (4°C) for 10 min at 5,000 g, pellets were washed once in sterile MilliQ water and centrifuged. The pellets were re-suspended in 0.05% SDS by vortexing, centrifuged (4°C) for 10 min at 5,000 g and subsequently washed three times with sterile MilliQ water and stored at 4°C. The purity was checked by determining the colony-forming units (CFUs) of spore stocks that were unheated and those heated at 65°C for 1 h prior to growth on LB plates overnight at 37°C, and was typically >75%. As well as strains, spores used for kinetic experiments were verified by PCR (for example, Supplementary Figure S5) after germination in LB overnight at 37°C, using the primers listed in Supplementary Table S2.

### *Bs*CotA activity of purified spores

For kinetic measurements of *Bs*CotA activity, purified spores from the WT, Δ*csp3* and complemented Δ*csp3* strains were diluted with MilliQ water to give an absorbance at 580 nm of ∼1.2 (measured accurately), and heated at 65°C for 1 h prior to use. To determine the CFUs/ml for this suspension a 5 × 10^5^-fold dilution in LB was plated (100 μl) onto LB agar, incubated at 37°C overnight and colonies counted. An aliquot of the heat-treated spore suspension (100 μl) was added to 900 μl of 100 mM citrate-phosphate buffer pH 4.0 plus 0.1–2.4 mM ABTS, and the absorbance at 420 nm (*ε* = 35,000 M^−1^cm^−1^) measured for 5 min at 37°C (Supplementary Figure S3). A control using 100 μl of buffer was also measured and showed no change in absorbance at 420 nm. The initial velocity (*V*_0_; typically reported in units of μM/min/CFUs/mL) was calculated, and plots of *V*_0_ against ABTS concentration (Figure 2) were fit to the Michaelis-Menten equation to determine *V*_max_ (the maximum rate) and *K*_M_ (the Michaelis constant). Comparing *V*_max_ values calculated based on the absorbance at 580 nm of the heat-treated spore suspension, rather than using CFUs/mL, has no significant influence on the outcome of the study, but generally produces data with larger errors.

### Cloning and purification of *Bs*CotA

The *cotA* gene was amplified from *B. subtilis* genomic DNA using primers CotA_1F and CotA_1R listed in Supplementary Table S2, and cloned into pGEM-T. After removing the NdeI site in the gene by QuickChange site-directed mutagenesis (with primers CotA_2F and CotA_2R, Supplementary Table S2), the product was excised with NdeI and BamHI and re-cloned into pET11a. *Bs*CotA was overexpressed in *E. coli* BL21 (DE3) (100 μM IPTG) grown at 20°C for 24 h. The protein was purified using a modified version of a published procedure (Martins et al., 2002). Cells from 0.5 to 2.0 L of culture were resuspended in 20 mM Tris pH 8.5, sonicated and centrifuged at 40,000 g for 30 min. The supernatant was diluted five-fold in 20 mM Tris pH 8.5 (sometimes plus 1 mM EDTA) and loaded onto a HiTrap Q HP column (1 or 5 ml) equilibrated in the same buffer. Proteins were eluted with a linear NaCl gradient (0–500 mM, total volume 50–200 ml) and fractions analysed using 18% SDS-PAGE. *Bs*CotA-containing fractions were diluted with 20 mM Tris pH 7.6 (sometimes plus 1 mM EDTA) and loaded onto a HiTrap SP HP column (5 ml) and eluted with a linear NaCl gradient (0–500 mM, total volume, 200 ml). In some cases the *Bs*CotA-containing fractions were heated at 70 °C for 30 min [*Bs*CotA is a highly thermostable enzyme (Martins et al., 2002)], centrifuged at 40,000 g for 30 min, and the supernatant exchanged into 20 mM 4-(2-hydroxyethyl)piperazine-1-ethanesulfonic acid (HEPES) pH 7.5 plus 200 mM NaCl for further purification on a Superdex 75 10/300 GL gel-filtration column. Purified *Bs*CotA had no detectable Cu (<0.2 equivalents) associated with it when analysed by AAS (Vita et al., 2015; Vita et al., 2016), and showed minimal ABTS oxidation activity (<0.3 μmol/min/mg).

### Purification of *Bs*CopZ and sample preparation

*Bs*CopZ was purified as described previously (Vita et al., 2016) and contains a small amount of bound Zn(II). Samples were therefore incubated with >10 equivalents of EDTA for 1 h and exchanged with 20 mM HEPES pH 7.5 plus 200 mM NaCl. The resulting protein had no Zn(II) associated with it and was reduced with DTT under anaerobic conditions and desalted as described previously (Vita et al., 2015; Vita et al., 2016).

### Analysing the Activation of Recombinant apo-*Bs*CotA by Cu(I)-*Bs*Csp3 and Cu(I)-*Bs*CopZ

*Bs*Csp3 plus ∼18 equivalents of Cu(I) was prepared by adding the appropriate amount of a buffered solution of Cu(I) in an anaerobic chamber (Belle Technology, O_2_ << 2 ppm) to apo-protein in 20 mM HEPES pH 7.5 plus 200 mM NaCl, quantified using the 5,5′-dithiobis (2-nitrobenzoic acid) (DTNB) assay carried out in the presence of ∼6.0 M guanidine hydrochloride (Vita et al., 2015; Vita et al., 2016). Apo-*Bs*CotA was quantified using the absorbance at 280 nm (*ε* value of 84,739 M^−1^cm^−1^, Durão et al., 2008) and the number of free thiols determined with the DTNB assay (Vita et al., 2016). To reduce the Cys229-Cys322 disulfide (see Figure 1C), apo-*Bs*CotA was incubated overnight in the anaerobic chamber with a 100-fold excess of DTT. The protein was desalted twice on a PD10 column and quantified from the absorbance at 280 nm, with thiols measured using the DTNB assay. Fully-reduced *Bs*CopZ was also quantified using the DTNB assay and loaded with ∼0.8 equivalents of Cu(I) under anaerobic conditions. Cu(I)-*Bs*Csp3 [∼3 μM binding ∼49–55 μM Cu(I)] was mixed with ∼1.1–1.3 μM of either as-isolated or reduced apo-*Bs*CotA, requiring ∼4.4–5.2 μM Cu(I) to occupy all four Cu sites. Control experiments in which apo-*Bs*CotA was incubated with a similar concentration (∼49–55 μM) of either Cu(I) or Cu(II) were also analyzed, as was a mixture of apo-*Bs*CotA (1.1 μM) plus apo-*Bs*Csp3 (3.1 μM). Cu(I)-*Bs*CopZ [∼50–53 μM binding ∼40–42 μM Cu(I)] was separately added to ∼1 μM apo-*Bs*CotA, requiring ∼4 μM Cu(I) to fill all Cu sites. Mixtures were incubated at room temperature in the anaerobic chamber (some experiments with Cu(II) were performed in air as was the reaction between the two apo-proteins) for up to 48 h. To measure activity, 10 μl of each mixture was added to 990 μl of aerated 100 mM citrate-phosphate buffer pH 4.0 plus 2.4 mM ABTS, and the absorbance at 420 nm measured for up to 5 min at 37°C (Figures 3A,B,E). A similar concentration of apo-*Bs*CotA was also incubated anaerobically with just buffer (20 mM HEPES pH 7.5 plus 200 mM NaCl) and the lack of activity is clear (Figures 3A,D).

After some transfer experiments with reduced apo-*Bs*CotA plus Cu(I)-*Bs*Csp3 mixtures were loaded onto a 1 ml HiTrap SP HP column and eluted with a linear NaCl gradient (0–1 M, total volume 12 ml). Fractions containing *Bs*CotA were combined, concentrated and a UV/Vis spectrum measured. This not only enabled protein quantification from the absorbance at 280 nm, but allowed Cu(II) occupancies of the T1 and T3 sites to be estimated from the absorbance at 600 (*ε* = 3,870 M^−1^cm^−1^) and 330 (*ε* = 3,639 M^−1^cm^−1^) nm, respectively (Durão et al., 2008). The total Cu content of *Bs*CotA after transfer was measured using AAS (Vita et al., 2015; Vita et al., 2016).

The removal of Cu(I) by BCS (∼2.5 mM) was analysed for the Cu(I)-*Bs*Csp3 [∼0.8–1.2 μM plus ∼18 equivalents of Cu(I)] samples used for activity experiments, both in the absence (folded *Bs*Csp3) and presence (unfolding conditions) of guanidine-HCl (6.64 M) (Vita et al., 2015; Vita et al., 2016). The absorbance increase at 483 nm due to formation of [Cu(BCS)_2_]^3-^ (*ε* = 12,500 M^−1^cm^−1^) (Badarau and Dennison 2011a) was measured over time at 22°C in 20 mM HEPES pH 7.5 plus 200 mM NaCl (Figure 3F).

## Data Availability

The raw data supporting the conclusions of this article will be made available by the authors, without undue reservation.
