# Supplementary material for: Copper delivery to an endospore coat protein of Bacillus subtilis
Source: Front Cell Dev Biol. 2022 Sep 5;10:916114. doi: 10.3389/fcell.2022.916114 (PMC9484137; doi:10.3389/fcell.2022.916114)
Supplement: Supplementary file 1 [file DataSheet1.docx]

Copper delivery to an endospore coat protein of *Bacillus subtilis*

Jaeick Lee, Rosemary A. Dalton and Christopher Dennison *

Biosciences Institute, Newcastle University, Newcastle upon Tyne, NE2 4HH, UK.

*** Correspondence:**Christopher Dennison
christopher.dennison@ncl.ac.uk

**SUPPLEMENTARY MATERIAL**

CONTENTS:

Supplementary Figure S1. The influence of added Cu on the growth of WT and Δ*csp3 B. subtilis* in LB.

Supplementary Figure S2. The influence of added Cu to LB on Cu accumulation by WT and Δ*csp3 B. subtilis*.

Supplementary Figure S3. The influence of Cu levels and *Bs*csp3 on *Bs*CotA activity in *B. subtilis* spores.

Supplementary Figure S4. Schematic representation of the genomes of *B. subtilis* strains used in this work showing where primers hybridise.

Supplementary Figure S5. PCR analyses of the *B. subtilis* strains used in this study.

Supplementary Table S1. The removal of Cu(I) from *Bs*Csp3 by BCS over time.

Supplementary Table S2. Primers used in this study.


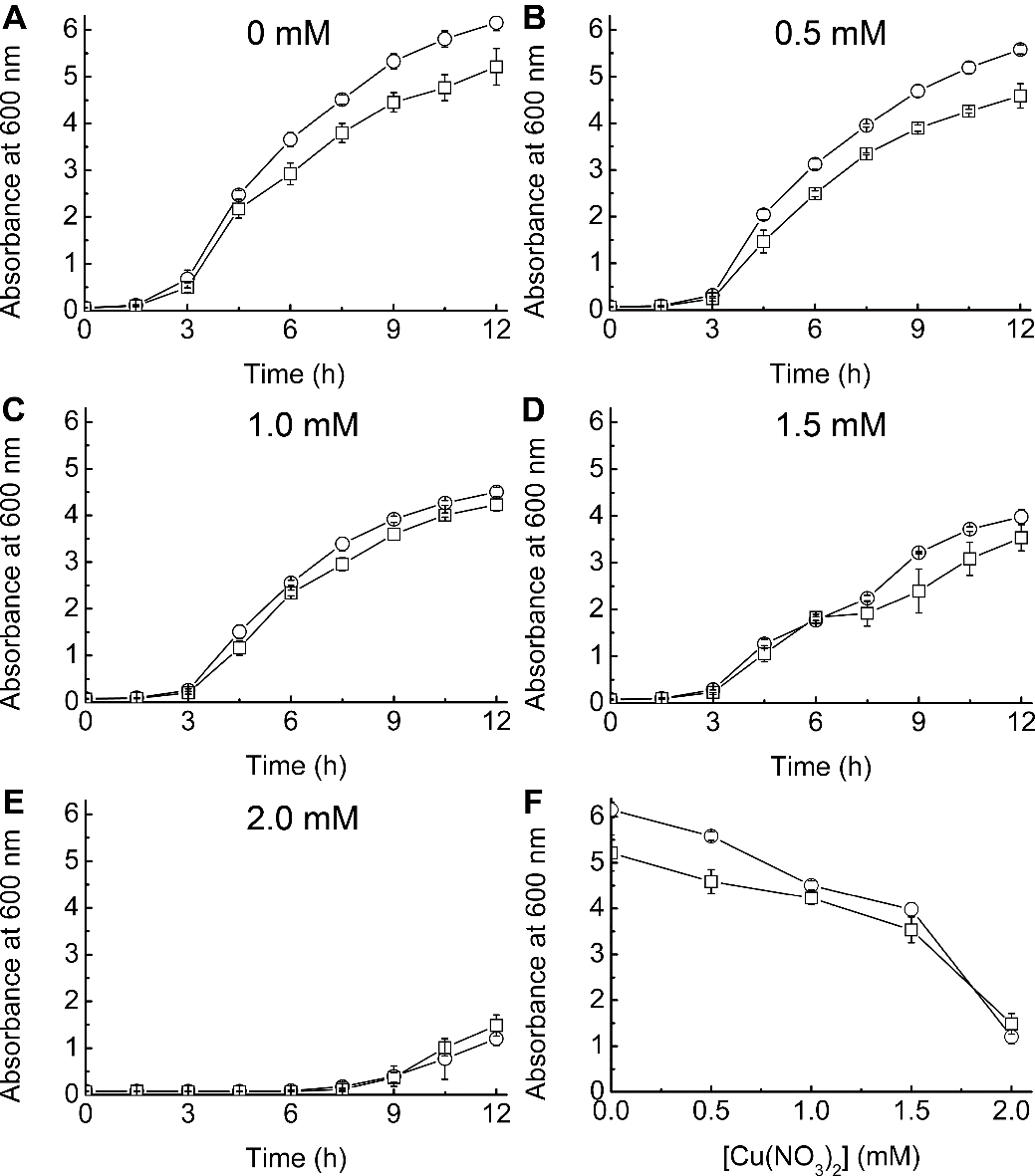


**SUPPLEMENTARY FIGURE S1.** The influence of added Cu on the growth of WT and Δ*csp3 B. subtilis* in LB. Plots of absorbance at 600 nm against time for WT (circles) and Δ*csp3* (squares) *B. subtilis* in LB plus 0 **(A)**, 0.5 **(B)**, 1.0 **(C)**, 1.5 **(D)**, and 2.0 **(E)** mM added Cu(NO_3_)_2_ grown at 37°C. All experiments were started from 100-fold diluted overnight cultures that had initial absorbance values at 600 nm of ~0.06-0.09. The data obtained at 12 h is compared in **(F)**, and in all cases averages and standard deviations from three independent growth experiments are shown. These results are similar to those we reported previously (Vita et al. 2016), particularly up to 12 h. However, the influence on growth started to be observed at a Cu(II) concentration in the medium that is ~0.5 mM lower in the earlier study. In the present work, we are sure of the Cu levels having carefully quantified all stock solutions by AAS, but did not do this previously (Vita et al. 2016). The absorbance of cultures were also measured at 24 h, and at up to 1.0 mM added Cu(NO_3_)_2_ a significant decrease was observed compared to the value at 12 h for both strains (consistent with previous data (Vita et al. 2016)). At higher added Cu(NO_3_)_2_ concentrations, the growth data showed no consistent pattern beyond 12 h. This differs to the enhanced cell death that was seen previously for Δ*csp3* *B. subtilis* after 12 h growth at reported medium Cu concentrations of 1.5-2.0 mM (Vita et al. 2016).


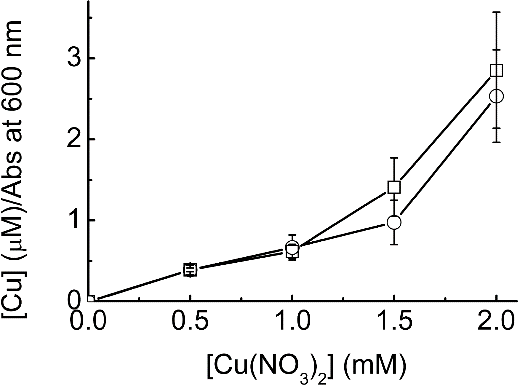


**SUPPLEMENTARY FIGURE S2.** The influence of added Cu to LB on Cu accumulation by WT and Δ*csp3 B. subtilis*. The intracellular Cu concentrations for WT (circles) and Δ*csp3* (squares) *B. subtilis* grown for 12 h in LB plus increasing amounts of added Cu(NO_3_)_2_. The data shown (average values and standard deviations) were measured for only two of the independent growth experiments, but the trend is clear.


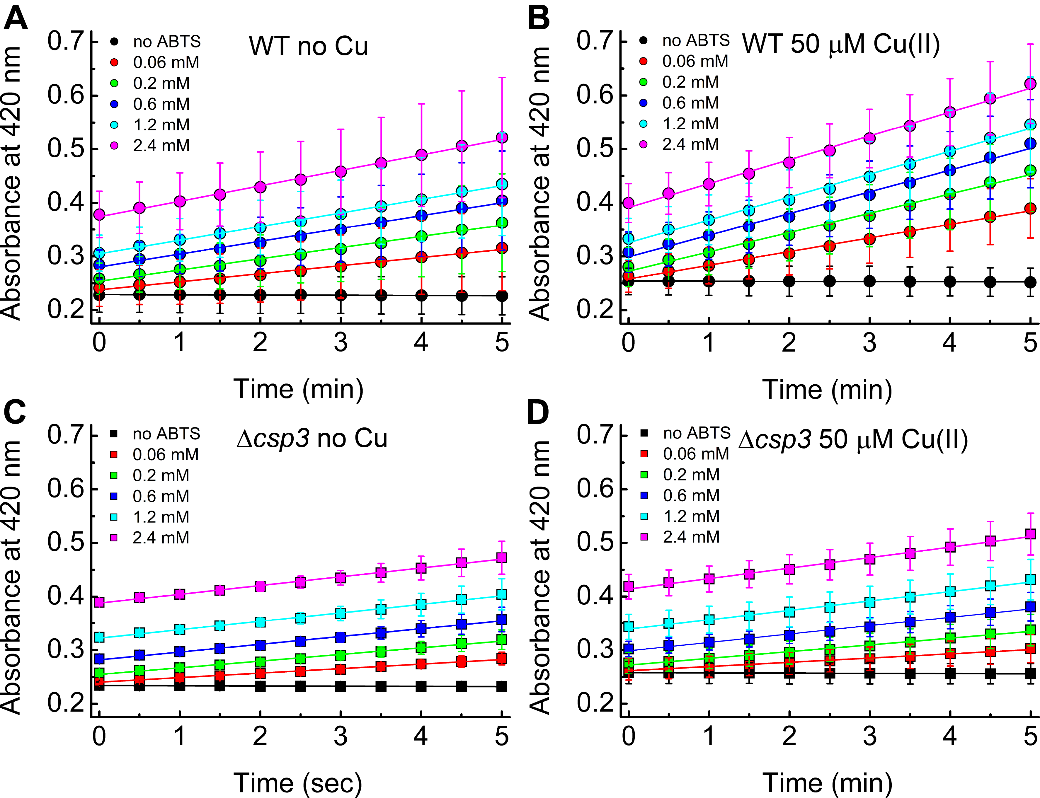


**SUPPLEMENTARY FIGURE S3.** The influence of Cu levels and *Bs*csp3 on *Bs*CotA activity in *B. subtilis* spores. Plots of absorbance at 420 nm against time at different concentrations of ABTS (indicated) for spores from WT **(A)** and **(B)** and Δ*csp3* **(C)** and **(D)** *B. subtilis*. The data in **(A)** and **(C)** are from spores obtained in DSM without added Cu, whilst 50 μM Cu(NO_3_)_2_ was added for **(B)** and **(D)**. The reactions with ABTS were measured in 100 mM citrate-phosphate buffer pH 4.0 and the initial rates (averages from three different sets of spores with error bars showing standard deviations) were used for **Figure 2A** (WT) and **Figure 2B** (Δ*csp3*).

**
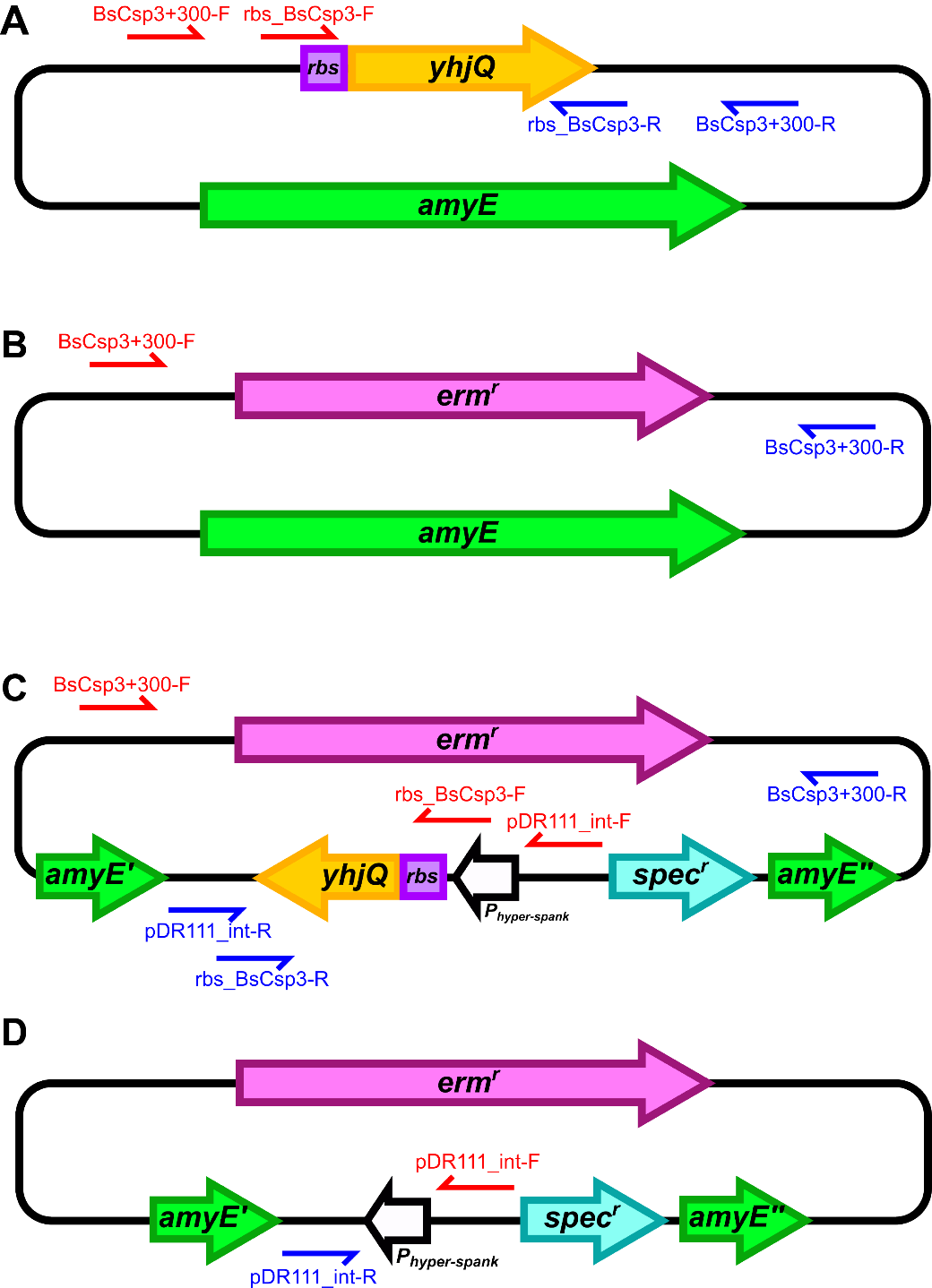
**

**SUPPLEMENTARY FIGURE S4.** Schematic representation of the genomes of *B. subtilis* strains used in this work showing where primers hybridise. Forward (red) and reverse (blue) primers for the PCR analysis (**Supplementary Figure S5 and Supplementary Table S2**) of **(A)** WT, **(B)** Δ*csp3*, **(C)** complemented Δ*csp3* and **(D)** a control strain generated by transforming Δ*csp3* *B. subtilis* with pDR111 are shown. The ribosome binding site (rbs) of the gene for *Bs*Csp3 (*yhjQ*), the erythromycin (*erm^r^*) and spectinomycin (*spec^r^*) resistance genes, and the α-amylase (*amyE*) gene are all labelled.

**
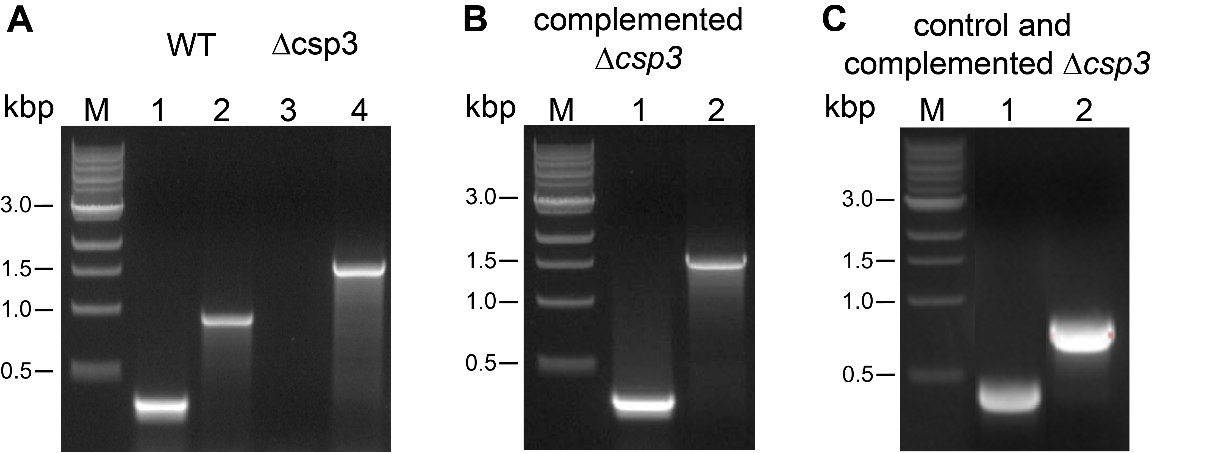
**

**SUPPLEMENTARY FIGURE S5.** PCR analyses of the *B. subtilis* strains used in this study. The rbs_BsCsp3-F/rbs_BsCsp3-R primers (lane 1) (**Supplementary Table S2**) give a product of 373 bp for WT **(A)** and no band for the Δ*csp3* strain (lane 3), whilst BsCsp3+300-F/BsCsp3+300-R give fragments of 967 and 1663 bp for WT (lane 2) and Δ*csp3* (lane 4), respectively. **(B)** Analysis of the complemented Δ*csp3* strain with rbs_BsCsp3-F/rbs_BsCsp3-R (lane 1) and BsCsp3+300-/BsCsp3+300-R (lane 2) give products of 373 and 1663 bp, respectively. The spores used for the three independent kinetic experiments on these strains **(Figure 2)** and gave the same results. Lane 1 of the gel shown in **(C)** contains the PCR product obtained for a control strain of Δ*csp3* *B. subtilis* transformed with pDR111 and analysed using the primers pDR111_int-F/pDR111_int-R (**Supplementary Table S2**) giving a fragment of 466 bp. Lane 2 shows the PCR product (831 bp) obtained when analysing the Δ*csp3* strain transformed with pDR111_rbs_csp3 (complementary Δ*csp3*) with the same primers. A molecular weight marker is included in lanes labelled with an M.

**Supplementary Table S1.** The removal of Cu(I) from *Bs*Csp3 by BCS over time.*^a,b^*

| Time (h) | % Cu(I) removal *^c^* |
| --- | --- |
| 0.5 | 12.9 ± 0.54 |
| 1 | 18.0 ± 0.71 |
| 2 | 24.7 ± 1.33 |
| 3 | 28.3 ± 1.23 |
| 4 | 31.5 ± 1.39 |
| 6 | 36.6 ± 1.50 |
| 17 | 52.9 ± 2.31 |
| 24 | 60.7 ± 2.35 *^d^* |

*^a^* Shown are the average percentage removal of Cu(I) from *Bs*Csp3 by BCS over time (and standard deviations) from three independent experiments.

*^b^* The amount of Cu(I) bound to the protein was determined by mixing the Cu(I)-*Bs*Csp3 sample with 2.5 mM BCS in the presence of 6.64 M guanidine-HCl in 20 mM HEPES pH 7.5 plus 200 mM NaCl, which unfolds the protein giving the maximum possible [Cu(BCS)_2_]^3-^ concentration (the value used is after incubation for 2 h).

*^c^* Percentage Cu(I) removal is determined using [Cu(BCS)_2_]^3-^ concentration/maximum [Cu(BCS)_2_]^3-^ concentration x 100.

*^d^* On one occasion the experiment was analysed up to 48 h with 70% Cu(I) removal observed.

**Supplementary Table S2.** Primers used in this study.

| Primers | Sequence |
| --- | --- |
| BsCsp3+300-F *^a^* | 5’-CATTCATGACAGTGCGACG-3’ |
| BsCsp3+300-R *^a^* | 5’-CACAAGAGGACTGGACGC-3’ |
| BsCsp3_seq-F *^b^* | 5’-CCGACAGCAGCAAATGCAGAAAACCA-3’ |
| BsCsp3_seq-R *^b^* | 5’-AAACCGTCGACTTTTACTTGCG-3’ |
| rbs_BsCsp3-F *^c^* | 5’-GGAGGACGC**AAGCTT**GCCGTGAAACATAAAACC-3’ |
| rbs_BsCsp3-R *^c^* | 5’-GGAGGACGC**GCTAGC**TTACGCTGCCATGCTGCGGC-3’ |
| BsCsp3-F *^d^* | 5’-GCGCATATGGAGCAATATTCTGAGGC-3’ |
| BsCsp3-R *^d^* | 5’-GCGCCATGGTTACGCTGCCATGCTGCGGC-3’ |
| pDR111_int-F *^e^* | 5’-GTGAACGCTCTCCTGAGTAG-3’ |
| pDR111_int-R *^e^* | 5’-GTCGGCTGAAAGATCGTAC-3’ |
| CotA-1F *^f^* | 5’-GGT**CATATG**ACACTTGAAAAATTTGTGGATGC-3’ |
| CotA-1R *^f^* | 5’-GGT**GGATCC**TTATTTATGGGGATCAGTTATATCC-3’ |
| CotA-2F *^g^* | 5’-CATTGACTTCACAGC**G**TATGAAGGAGAATCG-3’ |
| CotA-2R *^g^* | 5’-CGATTCTCCTTCATA**C**GCTGTGAAGTCAATG-3’ |

*^a^* Forward and reverse primers designed to hybridise ~300 bp upstream and downstream of the *csp3* gene giving PCR products of 1663 and 967 bp for Δ*csp3* and WT *B. subtilis*.

*^b^* Primers used to sequence the PCR product obtained from the Δ*csp3* strain using primers BsCsp3+300-F and BsCsp3+300-R.

*^c^* Forward (HindIII site in bold) and reverse (NheI site in bold, stop codon underlined) primers designed to amplify the *csp3* gene plus 28 bp at the 5’ end to include its own RBS for cloning into pDR111 and giving a product of 373 bp.

*^d^* Forward and reverse primers that amplify the *csp3* gene giving a PCR fragment of 345 bp.

*^e^* Primers that hybridise 294 bp upstream and 118 bp downstream of the region of pDR111 integrated into the *amy*E gene in the *B. subtilis* genome. For the complemented Δ*csp3* strain these give a 831 bp fragment, whilst in a strain transformed with just pDR111 the product is 466 bp.

*^f^* Forward (NdeI site in bold) and reverse (BamHI site in bold, stop codon underlined) primers designed to amplify the *cotA* gene.

*^g^* Forward and reverse primers used to mutate the NdeI site (from CATATG to CATACG, highlighted in grey with the site mutation in bold) within the *cot*A gene.
